# Total flavonoids of *Chrysanthemum indicum L* inhibit colonic barrier injury induced by acute pancreatitis by affecting gut microorganisms

**DOI:** 10.18632/aging.205924

**Published:** 2024-06-11

**Authors:** Xiaojuan Yang, Jia Hu, Chao Zhong, Song Xu, Shiyao Hua, Peng Liu, Ling He

**Affiliations:** 1 Department of Digestive System, Affiliated Hospital of Jiangxi University of Traditional Chinese Medicine, Nanchang 330000, Jiangxi Province, China

**Keywords:** colonic barrier dysfunction, gut microorganisms, TFC, Cerulein, acute pancreatitis

## Abstract

Background: Acute pancreatitis (AP) is a prevalent acute abdominal condition, and AP induced colonic barrier dysfunction is commonly observed. Total flavonoids of *Chrysanthemum indicum L* (TFC) have exhibited noteworthy anti-inflammatory and anti-apoptotic properties.

Methods: We established AP models, both in animals and cell cultures, employing Cerulein. 16S rRNA gene sequencing was performed to investigate the gut microorganisms changes.

Results: *In vivo*, TFC demonstrated a remarkable capacity to ameliorate AP, as indicated by the inhibition of serum amylase, myeloperoxidase (MPO) levels, and the reduction in pancreatic tissue water content. Furthermore, TFC effectively curtailed the heightened inflammatory response. The dysfunction of colonic barrier induced by AP was suppressed by TFC. At the *in vitro* level, TFC treatment resulted in attenuation of increased cell apoptosis, and regulation of apoptosis related proteins expression in AR42J cells. The increase of *Bacteroides sartorial*, *Lactobacillus reuteri*, *Muribaculum intestinale*, and *Parabacteroides merdae* by AP, and decrease of of *Helicobacter rodentium*, *Pasteurellaceae bacterium*, *Streptococcus hyointestinalis* by AP were both reversed by TFC treatment.

Conclusions: TFC can effectively suppress AP progression and AP induced colonic barrier dysfunction by mitigating elevated serum amylase, MPO levels, water content in pancreatic tissue, as well as curtailing inflammation, apoptosis. The findings presented herein shed light on the potential mechanisms by which TFC inhibit the development of AP progression and AP induced colonic barrier dysfunction.

## INTRODUCTION

*Chrysanthemum indicum L.* possesses a diverse range of pharmacological attributes, exhibiting properties such as broad-spectrum antibacterial, antiviral, anti-inflammatory, cardioprotective, and antioxidant effects [1, 2]. The key active constituents of *Chrysanthemum indicum L.* are the total flavonoids of *Chrysanthemum indicum L.* (TFC) [3]. Previous investigations have indicated that TFC exerts inhibitory effects on various pathogens including *Staphylococcus aureus, Escherichia coli*, and *diphtheria in vitro*. Furthermore, TFC has displayed anti-inflammatory properties in certain contexts [2].

Acute pancreatitis (AP) is a common and severe abdominal condition characterized by its rapid onset and potentially life-threatening consequences [4]. Apart from the localized pathological damage observed, it often triggers a robust systemic inflammatory cascade response, which can ultimately result in multiple organ failure and, in some instances, fatalities [5]. Clinical studies have reported a high mortality rate of up to 20% associated with AP, underscoring the vital clinical importance of controlling or mitigating severe acute pancreatitis [6].

Damage to the intestinal barrier function in AP can lead to the translocation of bacteria or endotoxins through various mechanisms, causing alterations in the intestinal flora and initiating enterogenous endotoxemia [7]. Secondary infections in pancreatic tissue can trigger systemic inflammatory response syndrome (SIRS), which may subsequently give rise to multiple organ dysfunction syndrome (MODS) [6]. Preserving the integrity of the intestinal barrier function is crucial as it plays a significant role in maintaining the body’s internal environment, reducing inflammatory reactions, and preventing the translocation of pathogenic bacteria and toxins from the intestines into the bloodstream [8]. Research has provided evidence that TFC can enhance the immune function in immunosuppressed mice and exert antibacterial effects [9]. Furthermore, TFC has been shown to inhibit the production of inflammatory mediators. However, the specific mechanisms underlying its anti-inflammatory effects in the context of AP warrant further investigation.

In this study, we employed Cerulein to induce AP model, both *in vivo* and *in vitro*, to investigate the impact of TFC on alleviating AP. Notably, TFC treatment was found to effectively mitigate the inflammatory response and apoptosis associated with AP in the experimental models. This research offers valuable insights into the potential mechanism underlying the inhibitory effects of TFC on AP.

## RESULTS

### Inhibition of AP was achieved by TFC *in vivo*

**Figure 1:**
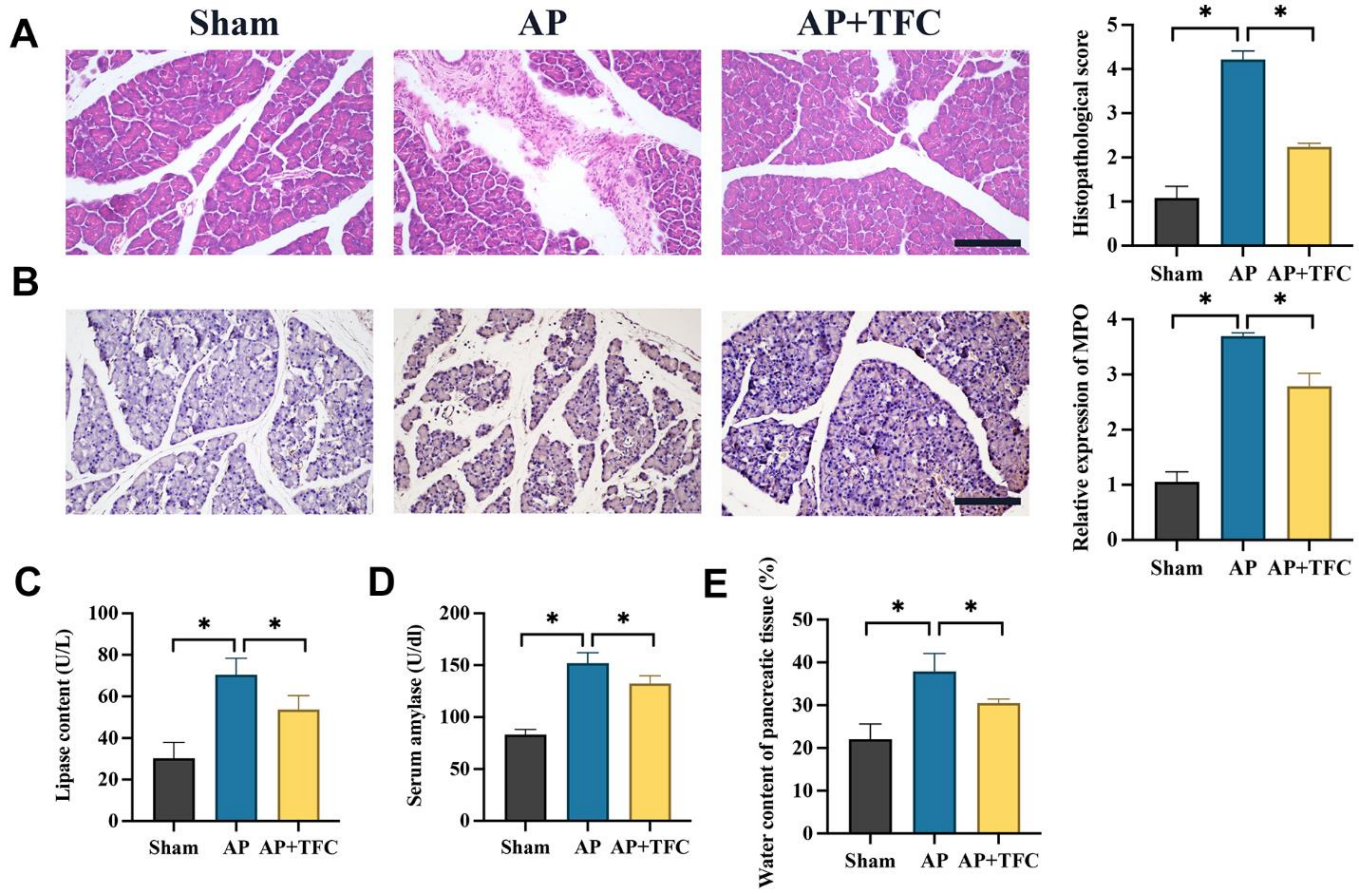
**Inhibition of AP *in vivo* was achieved by TFC.** (**A**) HE staining was performed to observe pancreatic tissue injury (Magnification 200X); (**B**) The level of MPO was measured with IHC staining (Magnification 200X); (**C**, **D**) The levels of lipase content and serum amylase were measured; (**E**) The water content of pancreatic tissue was calculated. * indicates p<0.05. Scale: 200 μm.

Histological examination using HE staining was employed to assess the impact of TFC on pancreatic tissue injury. In the sham operation group, pancreatic cells exhibited structural integrity with no signs of edema or inflammatory cell infiltration in the stroma. Conversely, the AP group displayed a substantial number of deceased pancreatic cells, accompanied by pronounced inflammatory cell infiltration and edema within the stromal tissue. Following TFC intervention, a marked reduction in both pancreatic cell death and inflammatory cell infiltration was observed (Figure 1A).

The significant increase of myeloperoxidase (MPO) in AP was inhibited by TFC (Figure 1B). Furthermore, in rats subjected to Cerulein treatment, there was a substantial elevation in the levels of lipase content (Figure 1C), serum amylase (Figure 1D), pancreatic tissue water content (Figure 1E) within the pancreatic tissue. However, treatment with TFC led to a pronounced inhibition of these parameters, indicating a potential protective role of TFC against AP-induced injury.

### The increased inflammatory response in AP were decreased after TFC treatment

**Figure 2:**
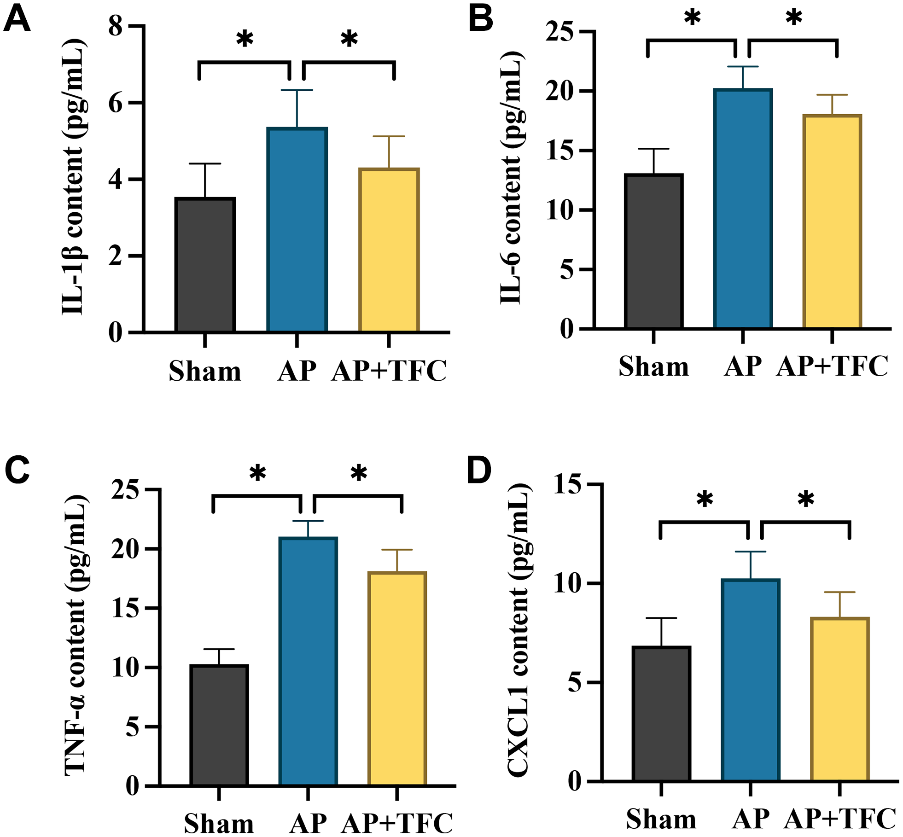
**The increased inflammatory response in AP were decreased after TFC treatment.** (**A**–**D**) The concentrations of IL-1β, TNF-*a*, IL-10, and CXCL1 in the serum were detected with ELISA method. * indicates p<0.05.

Elevated levels of inflammatory factors serve as crucial markers in AP. In the AP group, there was a significant increase in the expression levels of pro-inflammatory factors, notably IL-1β, TNF-*a*, IL-6, and CXCL1 (Figure 2A–2D). Notably, the alterations in the levels of these inflammatory factors observed in AP rats were effectively reversed by the administration of TFC.

### The dysfunction of colonic barrier induced by AP was suppressed by TFC

**Figure 3:**
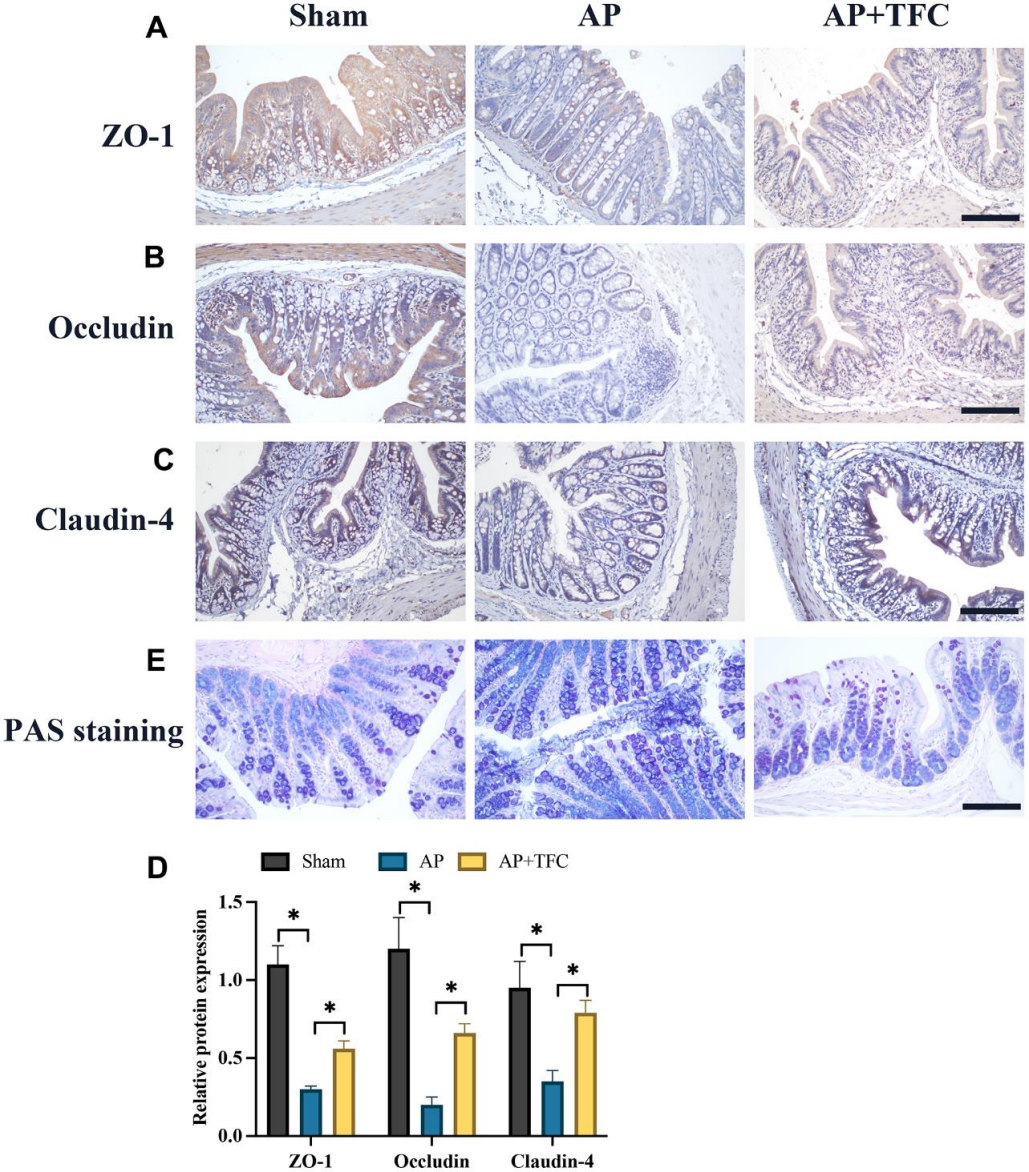
**The dysfunction of colonic barrier induced by AP was suppressed by TFC.** (**A**–**D**) The expression intensity of ZO-1, Occludin, Claudin-4, were measured with IHC staining (Magnification 200X); (**E**) PAS staining method was performed to detect intestinal mucus in tissues. * indicates p<0.05. Scale: 200 μm.

The dysfunction of colonic barrier induced by AP has been widely reported. Therefore, we investigate the influence of TFC in intestinal barrier function proteins, zonula occludens-1 (ZO-1), Occludin, Claudin-4, using IHC staining. We found that the expression of ZO-1, Occludin, Claudin-4 were markedly suppressed in the group AP (Figure 3A–3D). However, treatment with TFC greatly elevated the levels of ZO-1, Occludin, and Claudin-4. PAS staining method is mainly used in histology to detect sugars and intestinal mucus in tissues. We found that the decreased intestinal mucus in tissues in the group AP was greatly increased by TFC (Figure 3E).

### The augmentation of cell apoptosis and the upregulation of inflammatory factors *in vitro* were effectively attenuated by TFC

**Figure 4:**
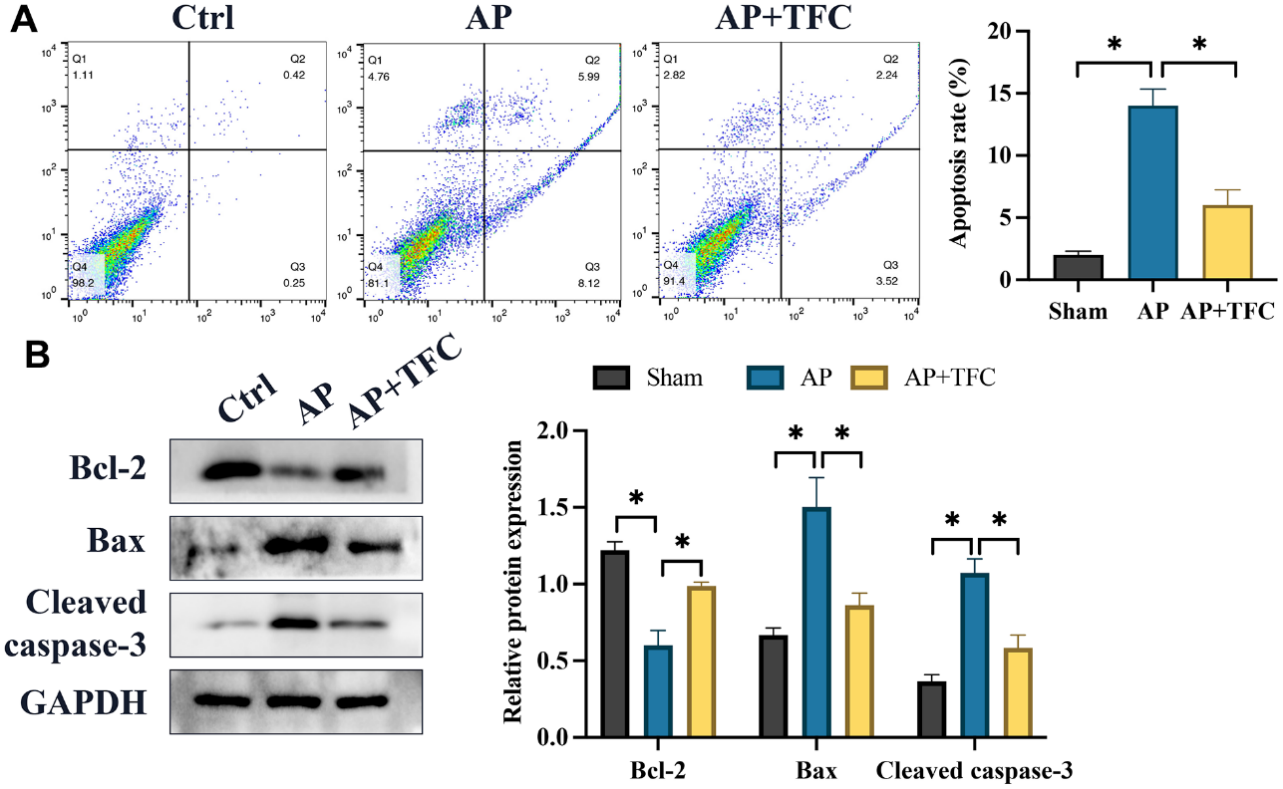
**The augmentation of cell apoptosis and the upregulation of inflammatory factors *in vitro* were effectively attenuated by TFC.** (**A**) Cell apoptosis was assessed using flow cytometry; (**B**) Proteins associated with apoptosis were quantified through Western blot analysis. * indicates p<0.05.

Cerulein was employed to treat AR42J cells to establish an *in vitro* model simulating AP. Following Cerulein treatment, a substantial increase in cell apoptosis (Figure 4A) and the expression of pro-apoptotic genes, namely Bax and cleaved caspase-3, was observed, whereas B-cell lymphoma 2 (Bcl-2) showed a remarkable decrease (Figure 4B) compared to the control group. However, treatment with TFC significantly counteracted the effects of Cerulein, leading to a suppression in the rate of cell apoptosis (Figure 4A). Furthermore, the changes in the expression levels of cell apoptosis-related proteins instigated by Cerulein were also effectively reversed by TFC (Figure 4B).

### TFC regulates the gut microorganisms of AP rats

**Figure 5:**
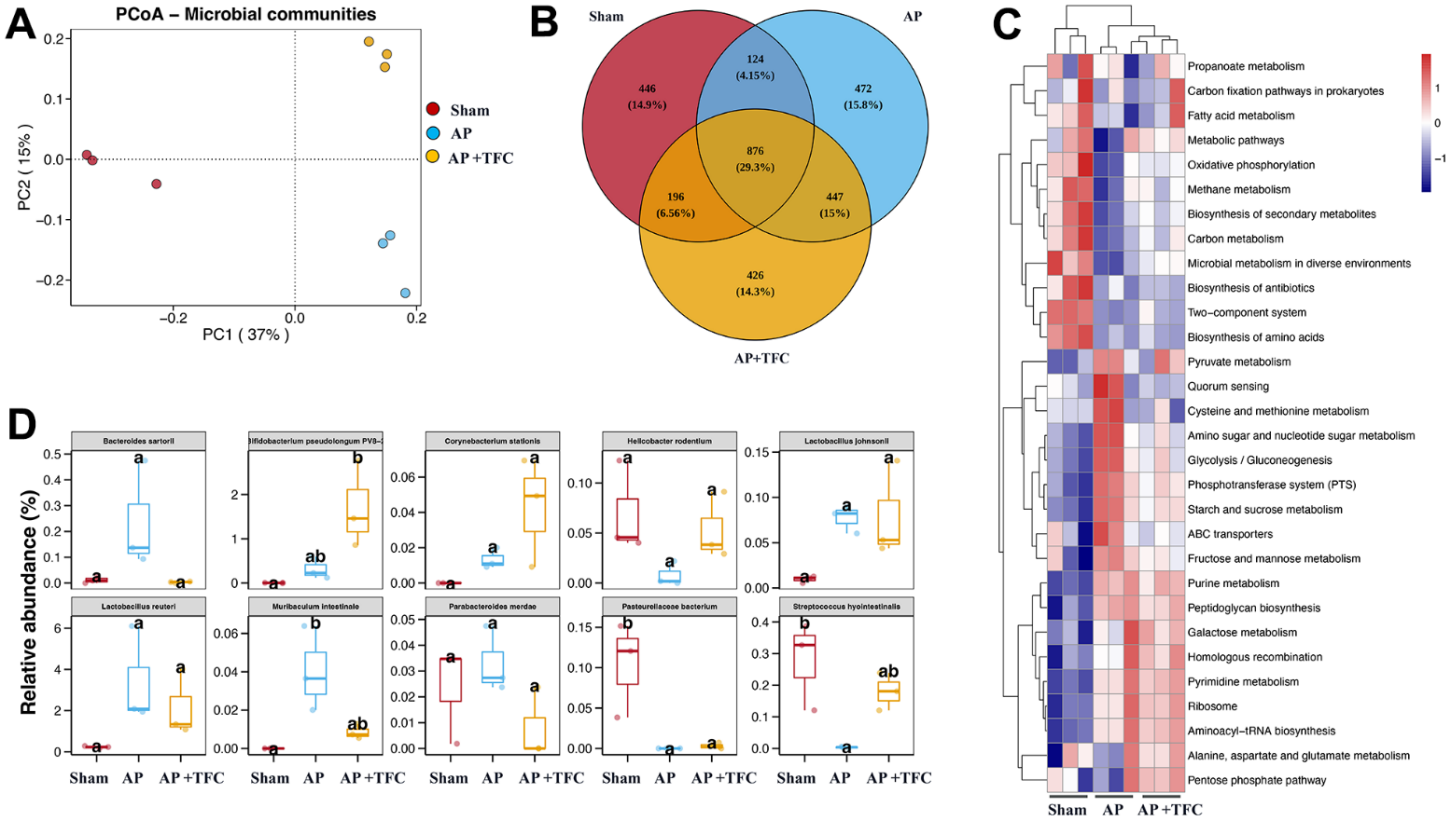
**TFC regulates the gut microorganisms of AP rats.** (**A**) PCoA analysis was performed; (**B**) core OTUs Venn was performed; (**C**) Clustering heat map was performed to analyze gut microorganisms difference; (**D**) Top 10 difference gut microorganisms were analyzed.

PCoA analysis is used to study the similarity or difference in the composition of sample communities. The data indicates that there is little difference within the group, and there are certain differences between the groups (Figure 5A). The number of bacterial species, genera, etc. in the sample sequencing results was obtained in inter group core OTUs Venn diagram (Figure 5B). The top 10 difference gut microorganisms were observed among different groups (Figure 5C, 5D). The levels of *Bacteroides sartorial*, *Lactobacillus reuteri*, *Muribaculum intestinale*, and *Parabacteroides merdae* were greatly increased in the group AP compared with group Sham. Meanwhile, the levels of *Helicobacter rodentium*, *Pasteurellaceae bacterium*, *Streptococcus hyointestinalis* were greatly decreased in the group AP compared with group Sham (Figure 5C, 5D). However, treatment with TFC significantly reversed the influence of AP on gut microorganisms.

## DISCUSSION

AP is a frequently encountered clinical condition characterized by acute abdominal symptoms and is classified under SIRS [10]. AP is often associated with bacterial translocation resulting from the disruption of the intestinal mucosal barrier [11]. Studies have indicated that damage to the intestinal mucosal barrier function, attributed to microcirculatory disorders, ischemia-reperfusion injuries, excessive release of inflammatory mediators, and increased apoptosis, can exacerbate the severity of AP [12]. In this research, we demonstrated that TFC remarkably inhibited the inflammatory response (Figure 2) and dysfunction of intestinal barrier (Figure 3) in the AP rats.

Recent pharmacological investigations have unveiled its broad-spectrum antibacterial properties. Additionally, studies have reported the significant anti-inflammatory and analgesic effects of TFC [13]. However, the precise mechanisms through which TFC regulates the progression of AP have yet to be fully elucidated. We present the novel findings that TFC can ameliorate AP by restraining inflammation and apoptosis. In our research, we observed that TFC treatment effectively mitigated the significantly elevated apoptosis and inflammatory response in AR42J cells (Figure 4).

The progression of AP is intricately linked to apoptosis. Within the mitochondrial pathway, Bax holds a prominent role as an apoptosis-inducing factor, driving the process [14]. Conversely, Bcl-2 acts as an anti-apoptotic protein with multifaceted functions encompassing the regulation of apoptosis and autophagy [15]. Furthermore, Bcl-2 can modulate autophagy and apoptosis induced by oxidative stress [16]. Bcl-2 competitively binds to Bax, thereby neutralizing its effects and diminishing apoptosis [17]. Our findings indicate that TFC effectively curtailed pro-apoptotic proteins and augmented the presence of anti-apoptotic proteins in pancreatic tissues and AR42J cells. This observed inhibition of apoptosis by TFC presents a plausible mechanism underlying the suppression of AP.

AP can cause imbalances in various gut microbiota, including the decrease in the number and diversity of gut microbiota [7]. AP may lead to a decrease in the number of beneficial bacteria in the intestine and an increase in the number of pathogenic bacteria [18]. This imbalance may reduce the diversity of gut microbiota, making the gut more susceptible to bacterial infections [12]. AP may lead to a decrease in beneficial bacteria in the intestine, while also increasing the number of susceptible bacteria such as *Escherichia coli* [19]. This may increase the risk of bacterial infection [20]. Inflammation and pathophysiological changes in acute pancreatitis may lead to damage to the intestinal mucosa [21]. This can disrupt the intestinal barrier function and increase the risk of pathogen invasion [22]. In this research, the changes of gut microorganisms caused by AP were greatly influenced by TFC. *Muribaculum intestinale* was reported to induce adaptive immune responses during homeostasis, and was greatly increased after AP induction [23]. However, TFC significantly reduced the level of *Muribaculum intestinale*. *Helicobacter rodentium* is believed to cause gastric ulcers and is even considered a pathogenic factor in gastric cancer. However, the levels of *Helicobacter rodentium* were markedly suppressed in the AP intestine, which needs to be further explored.

Previously, we demonstrated that TFC could significantly inhibit AP through restraining serum amylase, MPO, water content of pancreatic tissue, inflammation levels, apoptosis, and NF-κB signaling pathway activation [2]. In this research, we mainly, focused the dysfunction alleviation of colonic barrier, and regulation of gut microbiota disturbance after AP by TFC. This study expands the research mechanism of TFC’s inhibitory effect on AP.

## CONCLUSIONS

At the *in vivo* level, our findings demonstrated that TFC exhibits significant ameliorative effects on pancreatic tissue injury. It effectively suppresses the elevation of serum amylase levels, reduces MPO expression, mitigates pancreatic tissue edema, curbs the inflammatory response. The dysfunction of colonic barrier induced by AP was suppressed by TFC. At the *in vitro* level, we have validated the ability of TFC to hinder cell apoptosis. This comprehensive investigation elucidates the potential mechanistic underpinnings of TFC in mitigating the development of acute pancreatitis induced colonic barrier dysfunction.

## MATERIALS AND METHODS

### Establishment of acute pancreatitis animal model

The induction of acute pancreatitis was achieved through intraperitoneal injection of Cerulein. A total of 45 Sprague-Dawley rats were randomly allocated into three distinct groups, namely the sham group, AP group, and AP+TFC group. Prior to induction, all rats underwent a 12-hour fasting period, which was maintained for an additional 6 hours after the procedure. Following laparotomy, the group designated as sham underwent no further interventions and the intestinal and biliary pancreatic ducts were gently repositioned. In the AP group, rats were intraperitoneally administered Cerulein (50 μg/kg, #HY-A0190 MedChemExpress, USA) in seven consecutive doses, with a 1-hour interval between each administration. Subsequently, in the AP+TFC group, rats were subjected to TFC treatment (300 mg/kg) via oral gavage, with a frequency of once every 12 hours, and this regimen continued for three consecutive days after Cerulein induction as described previously [2]. Rats in the sham and AP groups received equivalent volumes of normal saline. Ultimately, all rats from each group were humanely euthanized for the collection of tissue and blood samples, with peripheral blood samples obtained through the removal of the eyeballs.

### Cell culture and AP cell model establishment

Rat pancreatic acinar cells, specifically AR42J cells (#CRL-1492, ATCC, USA), were employed. These cells were cultured within 5 cm × 5 cm cell culture bottles, allowing for adherence to the culture surface. The culture medium utilized for these cells was RPMI 1640, supplemented with 10% Fetal Bovine Serum (FBS, SH30071.03IR25-40, Hyclone, Cytiva, USA) and Antibic Antimycotic double antibody. The culture conditions were maintained at a temperature of 37° C with a CO_2_ concentration of 5%. A total of 1 × 10^6^ cells were seeded in individual wells of a 6-well cell culture plate and were allowed to incubate for a period of 24 hours. Subsequently, Cerulein was introduced into the culture medium at a final concentration of 10 nmol/L. After a 4-hour incubation period, TFC (50 mg/L) was added to the cell cultures, where it remained for a duration of 24 hours. The cells in the control group were treated with same amount of PBS. Following this, the supernatant from the cell culture, as well as the AR42J cells themselves, were meticulously collected and reserved for subsequent experimental procedures.

### Flow cytometry

Following the cell culture and incubation, a total of 1 × 10^5^ cells were harvested and subsequently subjected to centrifugation at a rate of 800 rpm, accomplished at room temperature and lasting for 5 minutes, effectively separating the cellular supernatant. These cells were then subjected to two cycles of PBS wash. To prepare the cells for analysis, 500 μL of a binding buffer was introduced. Within this medium, 5 μL of Annexin V–FITC was suspended in the cellular mixture and allowed to remain in the absence of light, at room temperature, for a duration of 15 minutes. Following this incubation period, 10 μL of propidium iodide (PI, Beyotime, #C1062L, China) was subsequently added. The state of cell apoptosis was ultimately assessed via the utilization of flow cytometry, enabling a comprehensive examination of the cellular response.

### Hematoxylin and eosin (HE) staining

The freshly procured pancreatic tissue from mice was subjected to fixation in formalin solution for an extended period of 24 hours. The obtained pathological sections were allowed to desiccate thoroughly overnight at a temperature of 60° C. Subsequently, the sections were subjected to a dewaxing process using xylene. A progressive hydration protocol was then administered, entailing sequential rinsing with 100%, 95%, 75%, and 50% ethanol solutions. Following this, the sections were immersed in PBS for a period of 3 minutes. Hematoxylin was applied for a duration of 3 minutes to effectively stain the cell nuclei, with subsequent rinsing using tap water. A brief 10-second immersion in a 75% hydrochloric acid ethanol differentiation solution was carried out, followed by another round of tap water washing for a span of 3 minutes. The sections were further soaked in 95% ethanol for 2 minutes and subjected to eosin staining for 30 seconds. Dehydration of the slides was then facilitated using a series of ethanol solutions at concentrations of 75%, 95%, and 100%. Transparency was conferred upon the sections through a 5-minute immersion in xylene. Upon completion of these procedures, the sections were sealed with neutral gum and subsequently dried at 60° C overnight. The sections were then made ready for microscopic observation.

### Immunohistochemistry (IHC) staining

The preparation of slides was executed in accordance with the procedures detailed in section 2.4. Subsequently, the sections underwent antigen retrieval through a 3-minute microwave oven treatment. To block endogenous peroxidase activity, the sections were exposed to a 5% hydrogen peroxide solution. Following a thorough PBS wash, the slides were subjected to a 20-minute treatment with 5% BSA. Thereafter, the sections were incubated with the primary antibody, allowing the process to extend overnight at 4° C. Subsequent to a PBS rinse, the slides were exposed to the secondary antibody for a duration of 3 hours. DAB reagents (#P0203, Beyotime, China) were then applied to the slides, facilitating the visualization of sections. The sections were subsequently observed under a fluorescence microscope, with images recorded for analysis. The antibodies used in this research were listed as below: Rabbit monoclonal to ZO1 (#ab276131, Abcam, UK), rabbit monoclonal to Occludin (#ab216327, Abcam, UK), rabbit monoclonal to Claudin 4 (#ab210796, Abcam, UK).

### Detection of TNF-*a*, IL-6, IL-1β, CXCL1, and amylase

The levels of TNF-*a* (SEKR-0009, Solarbio, China), IL-6 (SEKR-0005, Solarbio, China), IL-1β (SEKR-0002, Solarbio, China), and CXCL1 (SEKR-0014, Solarbio, China) in the supernatant of AR42J cell cultures and the serum of rats were quantified using ELISA (Enzyme-Linked Immunosorbent Assay) techniques, following the manufacturer’s instructions provided with the kits, which were procured from Solarbio (Beijing, China). The level of amylase was measured with starch iodine colorimetric method (#C016-1-1, Nanjing Jiancheng Bioengineering Institute, Nanjing, China).

### Western blotting

Protein extraction from pancreatic tissues and cells was carried out, and their concentrations were determined using the BCA (Bicinchoninic Acid) method. Subsequently, SDS-PAGE (Sodium Dodecyl Sulfate-Polyacrylamide Gel Electrophoresis) was conducted to separate proteins by their molecular weight. The separated proteins were then transferred to a membrane. Blocking was performed using 5% skimmed milk at room temperature for 1 hour. A primary antibody (diluted at 1:1000) was applied and incubated with the membrane overnight at 4° C. The membrane was washed three times with TBST (Tris-Buffered Saline with Tween 20). Subsequently, an HRP (Horseradish Peroxidase) labeled secondary antibody (diluted at 1:2000) was added for a 2-hour incubation at 37° C. After another round of washing with TBST, protein bands were analyzed using ImageJ software. All antibodies used in this study were procured from Abcam. The antibodies used in this research were listed as below: rat monoclonal to Bcl-2 (#ab241548, Abcam, UK), rabbit monoclonal to Bax (#ab216985, Abcam, UK), rabbit polyclonal to Cleaved Caspase-3 (#ab2302, Abcam, UK).

### Periodic acid-Schiff (PAS) staining

The deparaffinization and hydration process was carried out to reach a hydrated state. Oxidation was then performed by immersing the samples in a 0.5% Periodic Acid solution for 5 minutes. Following this, the samples were rinsed in three changes of distilled water. They were subsequently placed in Schiff’s reagent for a duration of 15 minutes. A thorough washing step in tap water for 5 minutes was conducted, followed by counterstaining in Mayer’s hematoxylin for a period of 1 minute. The samples were then rinsed in tap water for another 5 minutes, with a final rinse in distilled water. The dehydration process was completed before covering the samples with coverslips and mounting them using a Xylene-based mounting medium.

### 16S rRNA gene sequencing

The genomic DNA from fecal samples was isolated utilizing the CTAB method, followed by an assessment of purity and concentration through 1% agarose gel electrophoresis. PCR amplification targeting the V3+V4 variable region was conducted using primers 341F (5’- CCTAYGGRGRBGCACAG - 3’) and 806R (5’- GGACTACNNGGGTATCTAAT - 3’) with the T100PCR instrument provided by Bio Rad Company in the United States. The resulting PCR products were mixed in equal concentrations based on their respective concentrations, ensuring thorough mixing. Subsequently, the PCR products were purified via agarose gel electrophoresis using a 2% TAE concentration, with the target bands excised and recovered. The Qiagen gel recovery kit from Qiagen Company (USA) was employed for PCR product recovery. The library construction was carried out using the TruSeq DNA PCR - Free Sample Preparation Kit Library Kit, a product of Illumina company ®. Quantification and evaluation of the constructed library were performed using Qubit, and after successfully passing the quality assessment, NovaSeq 6000 PE250 was employed for high-throughput sequencing on the Illumina platform. The sequencing process was conducted by Shenzhen Micro Science Alliance Biotechnology Co., Ltd., China

Bioinformatics analysis primarily involves the utilization of the QIIME2 dada2 software for quality control of sequencing results, encompassing tasks such as trimming, denoising, and concatenation, eventually leading to the acquisition of feature sequence tables post removal of chimeras. Subsequently, the representative sequences of Amplicon Sequence Variants (ASV) are compared with the pre-trained GREENGENES database (version 13_8, 99% similarity threshold) to generate a taxonomic classification table at the species level. To identify variations in gut microbiota abundance among groups and samples, various statistical methods are applied, including ANCOM, ANOVA, Kruskal Wallis, LEfSe, and DESeq2. Feature sequence-level α diversity indices, comprising observed Operational Taxonomic Units (OTUs), Chao1, and Shannon, are employed to assess sample diversity. On the other hand, β diversity indices such as Bray Curtis, unweighted UniFrac, and weighted UniFrac are utilized to evaluate distinctions in microbial community structure among samples. Lastly, the Redundancy Analysis method is deployed to explore potential correlations between microbial communities and pertinent environmental factors.

### Statistical analysis

Statistical analysis was conducted using SPSS version 22.0. Data were shown as mean values with standard deviations. The comparison of multiple groups was performed using ANOVA analysis, while independent sample t-tests were utilized to compare two groups. A significance level of p<0.05 was considered indicative of a statistically significant difference.

### Availability of data and materials

The datasets used in the current study are available from the corresponding author on reasonable request.
